# Impact of the introduction of ultrasound services in a limited resource setting: rural Rwanda 2008

**DOI:** 10.1186/1472-698X-9-4

**Published:** 2009-03-27

**Authors:** Sachita P Shah, Henry Epino, Gene Bukhman, Irenee Umulisa, JMV Dushimiyimana, Andrew Reichman, Vicki E Noble

**Affiliations:** 1 Department of Emergency Medicine, Alameda County Medical Center, 1411 E. 31st Street, Oakland, California, USA; 2 Department of Emergency Medicine, Zero Emerson #3B, Massachusetts General Hospital, 55 Fruit Street, Boston, Massachusetts, USA; 3 Division of Social Medicine and Health Inequalities, Brigham and Women's Hospital, 75 Francis Street, Boston, Massachusetts, USA; 4 Kirehe Hospital & Rwinkwavu Hospital, Partners in Health/Inshuti Mu Buzima (PIH/IMB), Eastern Province, Rwanda

## Abstract

**Background:**

Over the last decade, utilization of ultrasound technology by non-radiologist physicians has grown. Recent advances in affordability, durability, and portability have brought ultrasound to the forefront as a sustainable and high impact technology for use in developing world clinical settings as well. However, ultrasound's impact on patient management plans, program sustainability, and which ultrasound applications are useful in this setting has not been well studied.

**Methods:**

Ultrasound services were introduced at two rural Rwandan district hospitals affiliated with Partners in Health, a US nongovernmental organization. Data sheets for each ultrasound scan performed during routine clinical care were collected and analyzed to determine patient demographics, which ultrasound applications were most frequently used, and whether the use of the ultrasound changed patient management plans. Ultrasound scans performed by the local physicians during the post-training period were reviewed for accuracy of interpretation and image quality by an ultrasound fellowship trained emergency medicine physician from the United States who was blinded to the original interpretation.

**Results:**

Adult women appeared to benefit most from the presence of ultrasound services. Of the 345 scans performed during the study period, obstetrical scanning was the most frequently used application. Evaluation of gestational age, fetal head position, and placental positioning were the most common findings. However, other applications used included abdominal, cardiac, renal, pleural, procedural guidance, and vascular ultrasounds.

Ultrasound changed patient management plans in 43% of total patients scanned. The most common change was to plan a surgical procedure. The ultrasound program appears sustainable; local staff performed 245 ultrasound scans in the 11 weeks after the departure of the ultrasound instructor. Post-training scan review showed the concordance rate of interpretation between the Rwandese physicians and the ultrasound-trained quality review physicians was 96%.

**Conclusion:**

We suggest ultrasound is a useful modality that particularly benefits women's health and obstetrical care in the developing world. Ultrasound services significantly impact patient management plans especially with regards to potential surgical interventions. After an initial training period, it appears that an ultrasound program led by local health care providers is sustainable and lead to accurate diagnoses in a rural international setting.

## Background

In much of sub-saharan Africa, diagnostic imaging in patient care is limited to urban settings and lack of adequate health care facilities, personnel and diagnostic tools remain a major barrier to health-care delivery [1]. With non-governmental organizations efforts to strengthen and scale-up existing public sector health care models in rural international settings, attention has focused on appropriate placement of cost-effective, durable technology that will assist local health care providers in the clinical care of their patients.

In this study, we investigated the impact of a diagnostic ultrasound program on two rural district hospitals. Specifically, we evaluated which ultrasound applications were most commonly used in this setting, the accuracy of ultrasound image acquisition and interpretation post-training (ie after the physician trainer left and local physicians performed independently) and how the introduction of a diagnostic imaging modality such as ultrasound impacted patient mangagement plans and diagnoses. We hypothesize that ultrasound is sustainable in a low resource international setting, that ultrasound impacts patient management positively and can boost the diagnostic capacity of a rural center [2].

To our knowledge, few prior studies of ultrasound services in remote settings have ever been performed. In addition, questions regarding which patient populations would benefit most from this technology, which ultrasound applications are most useful, and how ultrasound impacts patient management plans and use of resources persist. Moreover, the sustainability of efforts to bring ultrasound imaging diagnostics to resource poor settings have never had long-term follow up results published regarding sustainability. We present answers to these questions based on our experience in rural Rwanda in 2008.

## Methods

Our study setting consisted of two rural hospitals located in the villages of Kirehe and Rwinkwavu in Eastern Province, Rwanda. These hospitals are affiliated with Partners in Health, a US-based nongovernmental organization that provides financial, educational and staff support for providing health care to a population of over 800,000 people in Rwanda.

Prior to beginning the ultrasound training program, a needs assessment was performed by reviewing hospital and clinic logbooks from 2007 to determine which ultrasound applications would likely address the diagnostic concerns of clinicians working with this patient population. Specifically, patient demographics, admission diagnosis, discharge diagnosis, prenatal clinic log information and maternity ward logbooks were reviewed. Results of this review suggested the major causes of patient visits were related to obstetric concerns, tuberculosis (TB), HIV, and malnutrition.

The ultrasound training curriculum spanned nine weeks and included lectures followed by practical hands-on scanning sessions. The curriculum was based on that used in emergency medicine residency programs in the United States to train in ultrasound and uses goal-directed ultrasound exams to answer specific questions. The curriculum was replicated at the two hospitals for all interested physician staff. A physician showing special interest and aptitude for ultrasound (as noted by the physician directing the ultrasound training) was chosen at each hospital to act as the local ultrasound coordinator for that site.

The first lecture covered principles of ultrasound physics, instructions on how to operate the machine, potential uses of ultrasound, an outline of the lecture series and training expectations, and image and data sheet recording instructions. The next lecture in the series covered obstetrical ultrasound including first trimester scanning basics (how to evaluate for ectopic or molar pregnancy), methods for estimating gestational age, and evaluation of fetal position, cervix and placenta. The cardiac ultrasound lecture covered evaluation for mitral stenosis, estimation of global left ventricular function and evaluation for pericardial effusion. These cardiac applications were chosen because of previous demographic work in this area showing a high prevalence of rheumatic heart disease, viral and HIV cardiomyopathy, and TB related pericardial effusions. Further lectures included hepato-biliary ultrasound including evaluation for ascites, cirrhosis, amebic abscess, echinococcal cysts, and cholecystitis, as well as renal ultrasound for evaluation of hydronephrosis and chronic kidney disease including HIV nephropathy. Advanced applications discussed were ultrasound evaluation of deep venous thrombosis and how to perform procedures including vascular access, abscess evaluation and drainage, and paracentesis and thoracentesis with ultrasound guidance.

Ultrasound training sessions were conducted by a fourth year emergency medicine resident with prior ultrasound experience (including obstetrical ultrasound) during residency training and credentialing certification as outlined by the American College of Emergency Physicians. The only exception was the cardiac ultrasound lecture which was given by a cardiologist from the United States working with Partners in Health to establish an ongoing heart failure program. The physician conducting the ultrasound training accompanied local physician staff on daily ward rounds using the ultrasound machine to demonstrate how to integrate bedside ultrasound into daily clinical practice.

A SonoSite Micromaxx (SonoSite Inc, Bothell WA) was used for the training and for the clinical studies during the post training period. At each hospital the machine had an endocavitary probe, a curved array abdominal probe, and cardiac probes for adult and pediatric patients. One hospital had a high frequency linear transducer as well.

Data sheets were completed for each ultrasound scan performed by the local care provider and included demographic information such as gender, age, HIV or TB status, indication for performing the exam, and scan type. Ultrasound operators also indicated whether the use of the ultrasound changed their management plan and, if so, what specific change was made. This information was entered into a Microsoft Excel 2007 (Microsoft Inc, Seattle WA) spreadsheet for data analysis.

After the initial training period, and departure of foreign staff, local providers continued to use the ultrasound and record their scans and interpretations. Hard copies of all scans from the 10 week post-training period were sent to the United States to undergo blinded review by an ultrasound fellowship trained emergency physician to assess ongoing accuracy and scan quality.

This study was approved by the sponsoring hospital's Institutional Review Board and the Rwandan National Ethics Committee.

## Results

### Demographics of Patient Population

Our experience in Rwanda suggests that the patient population likely to benefit most from the use of ultrasound in routine patient care are adult females [see Table 1]. Of adult females having ultrasound performed, 44% were pregnant at the time of ultrasound. While the HIV prevelance rate of this region is only 5.7%, patients undergoing ultrasound had an HIV positive rate of 19% and a TB positive rate of 13%, suggesting that patients suffering from these chronic diseases may also benefit from ultrasound [3].

**Table 1:** Demographics of patients receiving ultrasound

Total Patients = 242	Number	Percentage of total
Female	169	70%

Pregnant	74	44% of all Females

Adult	194	80%

HIV +	41	19%

TB +	32	13%

### Ultrasound Exam Types Commonly Used

**Figure 1:**
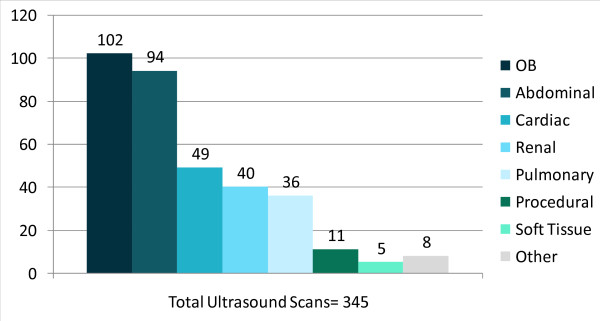
**Ultrasound scan types**. The most common ultrasound exam performed during routine clinical care was obstetrical ultrasound, followed closely by abdominal ultrasound.

There were many useful applications of ultrasound in this setting; however, obstetrical ultrasound, including estimation of gestational age, determining head position, and evaluating placental abnormalities, was the most frequently performed application overall [see Figure 1]. While both transabdominal and transvaginal approaches were used, transabdominal obstetric ultrasound was used more often (95 of 102 obstetric scans were transabdominal). This is most likely due to the fact that the majority of patients present for care for the first time in their 2^nd ^and 3^rd ^trimesters.

Non-obstetric abdominal indications for ultrasound included identification of ascites prior to paracentesis, assessment of liver disease such as cirrhosis, hepatic cysts or abcesses, abdominal tumor evaluation and biliary ultrasound. Because of the high burden of HIV related cardiomyopathies, TB-related pericardial effusions and rheumatic disease, basic cardiac ultrasound applications were also used quite frequently. Cardiac ultrasound included estimation of global left ventricular function, evaluation for mitral stenosis, and pericardial effusion evaluation. Renal ultrasound for assesment of hydronephrosis, chronic kidney disease and HIV nephropathy was used to help characterize which patients would benefit from costly interventions such as hemodialysis. Thoracic ultrasound for confirming and marking TB related pleural effusions prior to thoracentesis was another common application and benefited critically ill patients who were unable to travel from the wards to the radiography suite for their thoracentesis. This gave bedside clinicians more control over the treatment of their patients and allowed them to function more independently. Vascular ultrasound for deep venous thrombosis (DVT) evaluation in bedbound, hypercoagulable HIV patients and post-surgical patients was practice changing. As this diagnostic tool had not been previously available, a new anticoagulation management service was launched and included new access to heparin, warfarin, and diagnostic testing for INR. In two cases, the ultrasound was used to obtain emergency pediatric peripheral intravenous access after several failed attempts using conventional methods.

### Impact of Ultrasound on Patient Care

**Figure 2:**
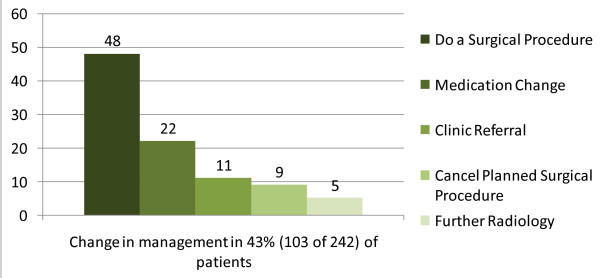
**Changes in clinical management based on ultrasound findings**. The use of the ultrasound changed patient management in 43% of cases. The most common change in management plan was to arrange a surgical procedure based on ultrasound findings; most commonly caesarean sections, biopsies of masses found, or minor surgeries.

Clinicians indicated on their datasheets that the ultrasound findings changed their initial patient management in 43% of patient cases, with the most common change indicated as a new plan to perform a surgical procedure after the ultrasound [see Figure 2]. Most often this surgical procedure was a cesarean section, biopsy, or minor surgery. For example, cesarean sections were performed based on ultrasound findings such as unexpected breech position of a fetus, placenta previa, or discovery of a multiple gestation pregnancy. Dilation & curettage procedures were performed after an ultrasound showed retained products of conception in females with vaginal bleeding. Other types of changes in patient care plan based on ultrasound results included medication changes such as adding furosemide for a patient with newly diagnosed heart failure by ultrasound, referrals to specialty clinic, cancelling planned surgical procedures such as paracentesis or thoracentesis if no fluid was identified by ultrasound, and referrals for further radiologic evaluation with computed tomography (CT) scanning.

### Sustainability

Sustainability of the ultrasound program was also evaluated. Clinicians continued to perform ultrasounds after the end of the ultrasound training curriculum and departure of foreign teaching faculty with almost twice the number of scans performed in the post training period as during the training period. In the 11 weeks post training, a total of 245 new scans were performed by the local staff at both hospitals with a similar distribution of application type and patient demographics. Previous studies of ultrasound in the developing world have raised concerns about the sustainability of maintaining the machines and upkeep of the technology. In our experience, the two Sonosite Micromaxx ultrasound machines have continued to function without any technical problems for the last 10 months. Ultrasound images from interesting or concerning cases are emailed regularly by the Rwandese staff physicians to the primary author of this paper for real-time quality assurance and assistance with interpretation.

### Quality and Accuracy of Ultrasound

Quality and accuracy of the ultrasound scans performed by the local staff after the training period were evaluated through blinded image review. An ultrasound-trained emergency physician blinded to the local staff sonographer's interpretation of the images reviewed a random selection of 112 scans from Kirehe Hospital from the 11 week post-training period. Of the 112 scans, 15 scans were considered technically limited studies due to missing views for adequate interpretation. Of the remaining 97 scans, 76 were considered true positive results, 18 true negative results, 3 false positive results, and 0 false negative results. Overall, the concordance rate of interpretation between the Rwandese physicians and the ultrasound-trained physicians doing quality review was 96%. Of the 3 false positive scans, the original interpretations were of an asymptomatic gallstone, small pericardial effusion, and LV global dysfunction. No patient management plans were changed by the false positive results.

## Discussion

Over the past several years, there has been a resurgence in world-wide interest and investment to bring new tools for the prevention, diagnosis and treatment of poverty-related disease to the world's poorest nations. This focus serendipitously coincides with improved ultrasound technology and machine engineering. As ultrasound technology becomes more portable, durable and affordable, it may become the diagnostic modality of choice in the developing world.

While the impact of ultrasound in the developing world is not well known, prior research has indicated that accurate point-of-care diagnosis using ultrasound could be useful in establishing timely diagnoses and early treatment in rural international settings. A recently published study by Spencer and Adler describes their experience as skilled American radiologists bringing a portable ultrasound for use on a medical mission in Ghana. They found that musculoskeletal ultrasound represented the most useful application in settings without plain film radiography access [4]. Their study confirms that ultrasound influenced the medical care of the patients in 40% of cases, but they do not address teaching local providers or establishment of a local ultrasound service after they finished their study. In one study from Sudan, Doehring-Schwerdtfeger et al reviewed the impact of ultrasound services implemented in a major urban hospital in 1986 and suggested that ultrasound was useful as a diagnostic aid in obstetrics, internal medicine and pediatrics, and changed patient management plans in 21% of patients [5]. A study by Steinmetz et al in 1999 shows general ultrasonography can be a useful diagnostic tool in rural Cameroon, with a high percentage of abnormal findings likely because ultrasound in this setting was used mainly to establish a positive diagnosis [6]. In this study, ultrasound contributed to a diagnosis in 61% of patients, which is similar to our findings. Our study confirms and elaborates on another small study performed in Butare, Rwanda by Mets in 1991, focusing on abdominal indications of ultrasound. These researchers found ultrasound aided diagnosis of cirrhosis, malignancy, renal disease, and and liver abcesses [7]. In another study of AIDS patients undergoing abdominal ultrasound in Central Africa, Tshibwabwa et al found AIDS patients had higher rates of ultrasound diagnosed hepato-splenomegaly, lymphadenopathy, biliary disease and ascites when compared with matched HIV negative controls [8]. Another prior study by Kobal et al focused solely on cardiac patients in rural Mexico. They suggested that hand carried ultrasound aids in diagnosis of valvular disease, congestive heart failure and congenital heart disease [9]. These findings are similar to ours. However, we also found several patients with TB related pericardial effusions; likely because of higher local prevalence rates.

## Conclusion

To our knowledge, this is the first study following the sustainability of an ultrasound program run by local health care providers in the developing world. In addition, there are few studies evaluating the change in management an ultrasound program in such a setting can effect. We suggest that ultrasound is a useful diagnostic tool that particularly benefits women's health and obstetrical care in the developing world. In addition, the use of ultrasound may impact patient management plans especially with regards to potential surgical interventions. Our research suggests that after an initial training period, an ultrasound program led by local health care providers can be sustainable and lead to accurate diagnoses in a rural international setting long after the instructing clinicians have departed.

Further study in this area is needed to gauge the diagnostic accuracy of ultrasound when performed by local health care providers months to years after completion of a training program. In addition, the ecominic impact of ultrasound services on resource utilization, institutional referral patterns and patient care in this type of setting needs further study. Longitudinal evaluation of this training program is ongoing.

## Competing interests

The authors declare that they have no competing interests.

## Authors' contributions

SS initiated the study design and carried out the ultrasound training, shared in the data entry and writing of the manuscript. HE contributed to the study design, oversight of data collection and editing of the manuscript. GB carried out the echocardiography training and arranged procurement of the ultrasound machines. IU and JD participated in data collection, data entry, and edited the manuscript. AR participated in data collection, data entry and statistical analysis. VN contributed to study design, training materials, and shared in editing and writing the manuscript. All authors read and approved the final manuscript.

## Pre-publication history

The pre-publication history for this paper can be accessed here:

http://www.biomedcentral.com/1472-698X/9/4/prepub
